# Risk factors for acute hepatitis B and its progression to chronic hepatitis in Shanghai, China

**DOI:** 10.1136/gut.2008.157149

**Published:** 2008-08-28

**Authors:** H W Zhang, J H Yin, Y T Li, C Z Li, H Ren, C Y Gu, H Y Wu, X S Liang, P Zhang, J F Zhao, X J Tan, W Lu, S Schaefer, G W Cao

**Affiliations:** 1 Department of Epidemiology, Second Military Medical University, Shanghai, China; 2 Department of Acute Infectious Diseases, Center for Disease Control and Prevention, Shanghai, China; 3 Department of Infectious Diseases, The 1^st^ Affiliated Hospital, Second Military Medical University, Shanghai, China; 4 Abteilung für Virologie, Universität Rostock, Germany

## Abstract

**Background and aims:**

The major risk factors for acute hepatitis B (AHB) in China and the viral factors determining the progression from acute to chronic hepatitis B remain largely unknown.

**Methods:**

Epidemiological studies within a population-based surveillance for AHB in adults were performed in Shanghai, China, including 294 patients, 588 matched controls and 572 family members of the patients.

**Results:**

Invasive medical procedures, household contact with hepatitis B virus (HBV) carriers, body care and beauty treatments, and lack of HBV vaccination were independently associated with AHB. Among those risks, pedicure in bath centres emerged. Sixty-eight of 128 patients with AHB were genotyped including 33 with HBV B2 and 35 with HBV C2. Twenty-five (8.50%) of the 294 patients, including 20 with HBV C2 and 5 with HBV B2 (p = 0.013), progressed to chronic infection. Multivariate analysis showed that HBV C2 was independently associated with chronicification of AHB. Patients with HBV B2 were younger and there was a higher proportion of women than those with HBV C2. The prevalence of HBV B2 was higher in the patients than in neighbourhood chronic carriers. The chronic carriers with HBV B2 showed higher viral loads, higher hepatitis B e antigen (HBeAg) seropositivity, and with higher proportion in men than those with HBV C2, implying that sexual contact plays a role in the transmission of HBV B2. Phylogenetic analysis showed that HBV C2 was frequently involved in transmissions within households.

**Conclusions:**

Despite lower viral load and HBeAg status in the chronic carriers, HBV C2 was more prone to causing chronic infection than was HBV B2.

Infection with hepatitis B virus (HBV) is a major global health problem. Approximately 3 billion people have been exposed to HBV, and more than 300 million are chronically infected with HBV.^1^ ^2^ In HBV non-endemic areas, most HBV infections are transmitted during adolescence or adulthood: sexual intercourse, use of injectable drugs, and nosocomial infection are risk factors for acute hepatitis B (AHB).^3^^–^5 Approximately 45% of the world’s population lives in regions where HBV infection is endemic.^2^ In the endemic areas, HBV infections frequently occur in the neonatal period or during early childhood, but very few studies on risk factors for AHB in adults have been reported.

In mainland China, an endemic area with one-third of the world’s HBV carriers, HBV transmission in the neonatal period used to be a major route.^6^ After nationwide HBV vaccination in newborns was implemented in 1992, the prevalence of HBV in children decreased dramatically. A nationwide survey in 2006 showed that the prevalence of hepatitis B surface antigen (HBsAg) was around 1.5% in children under the age of 8 years, and 7.18% in the nationwide population at an age between 1 and 59 years (unpublished data). In Shanghai, the annual incidence of AHB has been documented since 1993 (fig 1). Although an overall decrease is evident, the incidence of AHB remains high.

**Figure 1:**
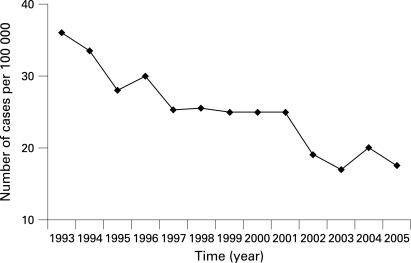
Annual incidence of reported cases of acute hepatitis B in Shanghai, China from 1993 to 2005. Approximately 17.78 million people were surveyed.

The natural course of HBV infection is very distinct. An important step in the natural course of HBV infection is the transition from acute to chronic infection. Induction of HBV chronicity has been shown to be influenced by the immune system and other host factors.^7^ ^8^ While newborns become chronic HBV carriers at a very high rate (about 90%), immune competent adults are generally described as developing chronic hepatitis at a rate of 5–10%.^1^ The natural course of HBV infection and the response to therapy are affected by HBV genotype.^9^^–^11 To date, eight genotypes (A–H) that differ by 8–15% at the nucleotide level have been identified.^12^ ^13^ HBV genotypes show distinct geographic prevalence.^12^^–^14 In Shanghai and surrounding areas, HBV genotypes C2 (62.1–72.9%) and B2 (13.4–28.0%) were the most prevalent genotypes in asymptomatic HBsAg carriers (ASCs), the patients with chronic hepatitis B and those with hepatocellular carcinoma.^15^ A contribution of viral genotypes to the persistence of HBV was suggested.^16^ However, no epidemiological study with a sufficient number of cases has shown an effect of HBV genotypes on the rate of HBV chronicity. In a limited number of cases, it was shown that HBV genotype D was associated with acute liver damage, while genotype A was associated with chronic outcome.^17^ ^18^

A study for the establishment of HBV infection in chimpanzees showed that dose-dependent experimental infection led to different outcomes.^19^ In a woodchuck model, high doses of woodchuck hepatitis virus induced high rates of chronicity, while chronicity rates were decreased for low doses of viral infection.^20^ In experimental studies conducted in the 1950s, patients who developed chronic hepatitis B had higher peak levels of viral markers that appeared early during the acute phase of infection than those with self-limited hepatitis.^21^ These observations imply that another potential viral determinant for the chronic outcome of AHB is the infection dose influenced by exposure type.

To elucidate the influence of HBV genotypes on the chronic progression of AHB, current risk factors for AHB, and its potential impact on the disease course, we conducted a community-based study in Shanghai, China.

## SUBJECTS AND METHODS

### Pilot study

To estimate the sample size for this 1:2 case–control study, an anonymous pilot survey of selected behaviours related to the transmission of HBV was initially conducted in Minhang District of Shanghai. In the population surveyed, exposure ratios of intravenous injection/infusion, pedicure, and beauty treatment were 12%, 14% and 5%, respectively. On the basis of the frequency of the exposures, an alpha level of 0.05, a beta level of 0.10, and crude odds ratio (OR) level of 2.5, it was calculated that sample sizes of 134 cases, 120 cases, and 275 cases were needed, respectively. In this study, 294 patients with AHB were estimated to provide statistically significant data.

### Cases and controls

Shanghai is composed of 18 administrative districts and one affiliated county. Five administrative districts including Songjiang, Baoshan, Pudong, Minhang and Nanhui were selected as study districts by a simple stochastic sampling method. AHB was diagnosed as follows: HBsAg and antibody to hepatitis B core antigen (anti-HBc) of the immunoglobulin M (IgM) class, acute-onset elevation (>45 U/l) of serum alanine aminotransferase levels. To discriminate AHB by new infection from acute exacerbation of chronic infection, the absence of serum HBsAg and anti-HBc before admission was verified by checking medical records. Previous negative HBsAg and anti-HBc status, together with the persistent presence of HBsAg in serum after an observation period of more than 6 months, was considered as progression to chronic infection.^22^ Patients with acute hepatitis A, hepatitis E, hepatitis C virus infection and/or co-infection, and drug- or alcohol-induced hepatitis were excluded. During January 2004 through December 2006, 298 AHB cases with medical records showing negative HBsAg were registered in the Centers of Disease Control and Prevention of five districts of Shanghai. Four of the 298 patients were excluded from the study because the controls did not match the cases.

Control subjects matched to the cases by sex and date of birth (within 2 years). A total of 624 candidate-matched controls were randomly recruited from the source population from which the cases arose at the time of AHB diagnosis. A total of 572 family members who had lived with the patient for more than 1 year were involved in the study. A total of 113 asymptomatic HBsAG carriers (ASCs) from the same source population were recruited during HBV screening, including 36 from the candidate-matched controls, 52 from the family members and 25 from the unmatched controls from the source population.

After giving written informed consent, residents who agreed to participate completed a questionnaire and provided blood samples.

### Epidemiological investigation

Patients with AHB and the controls were interviewed by trained research assistants using a standard, two-page questionnaire requesting information up to 6 months before the diagnosis of AHB. Information regarding sociodemographic characteristics, immunisation history, and exposures to known and potential risk factors for HBV infection was collected retrospectively. All participants were examined for HBV serological markers, including HBsAg, antibody to HBs (anti-HBs), hepatitis B e antigen (HBeAg), antibody to HBe (anti-HBe) and anti-HBc. Standard HBV vaccination with recombinant hepatitis B vaccine (Dalian Hissen Bio-Pharm, Dalian, China) was provided to those without HBV infection.

### Laboratory analysis

During the survey, 5 ml fasting blood from all participants was collected with a vacuum blood collection tube without anticoagulant. Serum samples of AHB patients during symptomatic phase were collected from hospitals. The serum was separated by centrifugation at 4°C and stored in a sterile tube at −80°C within 6 h of sample collection. Serological testing was performed as follows: HBsAg, HBeAg, anti-HBc immunoglobulin G (IgG), IgM, anti-HBs, anti-HBe, and antibodies to hepatitis C virus were examined by Architect-i2000 (Abbott Laboratories, North Chicago, Illinois, USA). Antibodies to hepatitis A virus and hepatitis E virus were examined by enzyme-linked immunosorbent assay (Abbott Laboratories).

HBV DNA was extracted from 200 μl sera by using the serum viral DNA purification kit (PG Biotechnology, Shenzhen, China). Serum viral load was measured in the LightCycler (Roche, Mannheim, Germany), using the Quantitative HBV PCR Fluorogence Diagnostic Kit (PG Biotechnology, Shenzhen, China). Genotypes of HBV were determined by using a multiplex polymerase chain reaction (PCR) assay developed in our laboratory^23^ and confirmed by DNA sequencing.

A part of the preS and S gene region of HBV genome was amplified by PCR and sequenced as described previously.^23^ The sequences from nt. 2919 to nt. 614 (910 base pairs) were isolated and registered in GenBank with accession numbers from EU216040 to EU216057. The sequences were aligned using the BioEdit (version 7.0.0) and CLUSTAL X 1.83 algorithms with default parameters. The phylogenetic tree was constructed by using the neighbour-joining component of the MEGA 4.0 computer program.^24^ Bootstrap analysis with 1000 replicates was used to determine the robustness of the tree and the evolutionary relationship of HBV.

### Statistical analysis

The data were entered (double entry) and analysed using the Statistical Program for Social Sciences (SPSS12.0 for Windows). The χ^2^ test was used to determine the differences in categorical variables. The odds ratio (OR) and 95% confidence interval (CI) for the factors under consideration were calculated in a univariate logistic regression analysis. To determine the factors contributing independently to AHB, forward stepwise multivariate regression analysis (p_entry_ = 0.05, p_removal_ = 0.10) was performed. The adjusted OR (AOR) for each risk factor was estimated by the adjustments with age, sex and educational level. Univariate and multivariate analyses were used to determine the factors including HBV genotypes, age and sex associated with chronicification of AHB. Serum viral load with skewed distribution was adjusted to normal distribution by transformation into logarithmic function, and then tested by the Student t test. A p value <0.05 was considered as statistically significant.

## RESULTS

### General information

**Figure 2:**
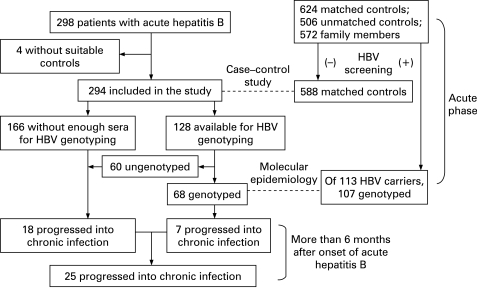
CONSORT flow diagram of this study. The broken lines (- - - -) indicate the type of epidemiological studies between the two population groups. HBV, hepatitis B virus.

The mean age of the 294 patients with AHB was 41.65 (SD 12.18) years and the ratio of men to women was 3.28:1. Thirty-six (5.77%) of the candidates matched 624 controls were excluded because of HBsAg seropositivity. Of 572 family members of AHB patients, 52 (9.09%) were found to be HBsAg seropositive. HBsAg seropositivity in the family members of AHB patients was higher than that of the matched controls (p = 0.028). HBV persisted in 25 (8.50%) of the 294 AHB patients. The CONSORT flow diagram is shown in fig 2.

A total of 78 patients with hepatitis A, 30 with hepatitis C, and 147 with hepatitis E were excluded, respectively. Of these patients, 40 with hepatitis A, 11 with hepatitis C, and 110 with hepatitis E were HBsAg seropositive.

### Risk factors for acute hepatitis B

In the univariate regression analysis, statistically significant ORs were obtained for household contact with chronic hepatitis B (p<0.001), liver cirrhosis (p<0.001), and hepatocellular carcinoma (p = 0.022), undergoing invasive medical procedures including intramuscular injection (p<0.001), intravenous injections or infusions (p<0.001), endoscopy (p = 0.024), and surgery (p = 0.019), body care and beauty treatment in public places including pedicure four or more times (p<0.001), and barber shop shaving four or more times (p = 0.008), eating out twice or more per week (p<0.001), and lack of HBV vaccination (p<0.001) in the patients with AHB (table 1). Piercing and tattooing in women (OR 2.06 (95% CI 1.11 to 3.81)), bleeding during pedicure (OR 4.29 (95% CI 1.92 to 8.55)) were also risk factors for AHB. No associations were suggested with the other reported risk factors, such as dental surgery, acupuncture, haemodialysis, blood transfusion, promiscuous sexual contact, being with a user of injectable drugs.

**Table 1:** Univariate and multivariate analyses of the risk factors exposed within 6 months before onset of acute hepatitis B: a community-based study

Potential risk factor	Cases	Controls	OR (95% CI)	AOR (95% CI)
Household contact with HBV carriers	101 (34.35)	89 (15.14)	2.93 (2.11 to 4.08)	3.05 (2.11 to 4.41)*
Spouse with HBV infection	21 (7.14)	25 (4.25)	1.73 (0.92 to 3.27)	
Mother with HBV infection	12 (4.08)	5 (0.85)	4.96 (1.73 to 14.22)	4.25 (1.11 to 16.22)†
Other members with HBV infection	45 (15.31)	24 (4.08)	4.23 (2.53 to 7.13)	3.64 (1.97 to 6.75)†
Family member with liver cirrhosis	18 (6.12)	5 (0.85)	7.60 (2.68 to 26.41)	4.04 (1.28 to 12.78)†
Family member with HCC	11 (3.74)	8 (1.36)	2.82 (1.04 to 7.76)	
Sharing razor	8 (2.72)	6 (1.02)	2.71 (0.82 to 9.57)	
Sharing towels	30 (10.20)	38 (6.46)	1.64 (0.97 to 2.79)	
Invasive medical procedure	103 (35.03)	77 (13.10)	3.56 (2.55 to 5.02)	3.72 (2.55 to 5.42)*
Surgery	12 (4.08)	9 (1.53)	2.74 (1.06 to 7.14)	
Endoscopy	10 (3.40)	7 (1.19)	2.92 (1.01 to 8.60)	
Intravenous injection or infusion	78 (26.53)	56 (9.52)	3.43 (2.31 to 5.10)	2.53 (1.52 to 4.21)†
Intramuscular injection	61 (20.75)	40 (6.80)	3.59 (2.29 to 5.62)	2.17 (1.22 to 3.84)†
Body care and beauty treatments in public places	130 (44.22)	155 (26.36)	2.21 (1.65 to 2.97)	1.52 (1.09 to 2.12)*
Barber shop shaving (⩾4 times)	58 (19.73)	76 (12.93)	1.66 (1.12 to 2.45)	
Receiving pedicure in the bath centre	103 (35.03)	102 (17.35)	2.55 (1.85 to 3.51)	1.98 (1.36 to 2.87)†
No	191	486		
Once	31	50	1.58 (0.95 to 2.61)	
Two to three times	21	28	1.91 (1.02 to 3.57)	
Four times or more	51	24	5.41 (3.15 to 9.33)	
Eating out (twice or more/week)	93 (31.63)	69 (11.73)	3.48 (2.41 to 5.02)	3.20 (2.14 to 4.77)*
Lack of HBV vaccination	285 (96.94)	484 (82.31)	6.80 (3.28 to 14.62)	7.78 (3.76 to 16.11)*

Data are expressed as number (%).

*Variables in the multivariate model included household contact with HBV carriers, invasive medical procedure, body care/beauty treatments in public places, eating out and lack of HBV vaccination.

†Variables in the multivariate model included mother with HBV infection, other members with HBV infection, family members with liver cirrhosis, intravenous injection or infusion, intramuscular injection, receiving pedicure in the bath centre, eating out, and absent HBV vaccination.

AOR, adjusted odds ratio; CI, confidence interval; HBV, hepatitis B virus; HCC, hepatocellular carcinoma; OR, odds ratio.

In multivariate regression analysis, household contact with a person or persons with liver cirrhosis (p = 0.018) and chronic hepatitis B (p<0.001), intramuscular injection (p = 0.008), intravenous injections or infusions (p<0.001), receipt of pedicure in bath centres (p<0.001), eating out twice or more per week (p<0.001) and lack of HBV vaccination (p<0.001) were independently associated with AHB.

In evaluating potential relationships among eating out and other risk factors, and their impact on AHB, eating out was frequently found to be related to the receipt of pedicure in the bath centres. In the patients with AHB, 53.8% of those eating out received pedicure in bath centres, while the percentage was only 34.8% in the controls (p = 0.017). As shown in table 2, receiving pedicure in bath centres after dinner parties played a role in HBV transmission.

**Table 2:** Effect of eating out and receiving pedicure services in the bath centres on acute hepatitis B

Eating out	Pedicure	Cases	Controls	OR (95% CI)
No	No	148 (50.3)	439 (74.6)	
	Yes	53 (18.1)	80 (13.6)	1.97 (1.70 to 2.97)
				
Yes	No	43 (14.6)	45 (7.7)	2.83 (1.75 to 4.59)
	Yes	50 (17.0)	24 (4.1)	6.18 (3.59 to 10.84)

Data are given as the number (%). CI, confidence interval; OR, odds ratio.

### Genotype distribution

**Figure 3:**
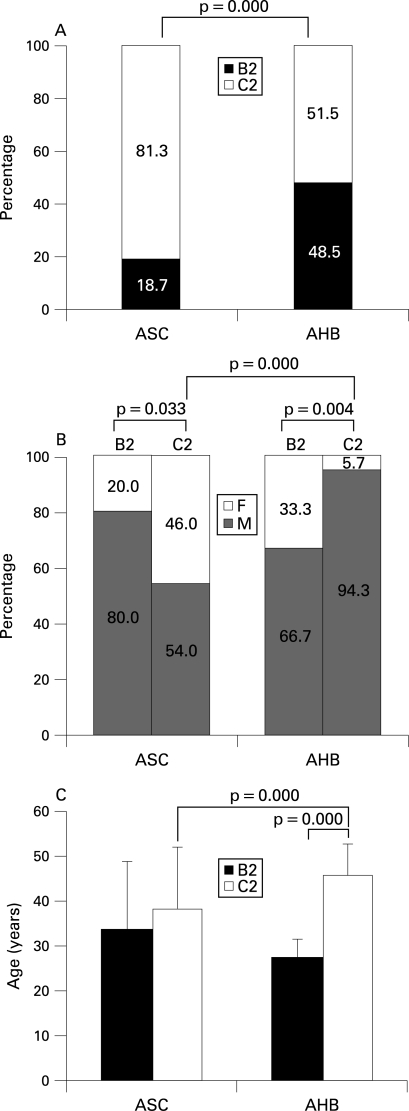
Distributions of genotypes (A), gender (B), and age (C) between 68 patients with acute hepatitis B (AHB) and 107 neighbourhood asymptomatic HBsAg carriers (ASCs). Age data were expressed as mean with the SD. B2, hepatitis B virus B2; C2, hepatitis B virus C2; F, female; M, male.

Serum samples of 128 patients with AHB were collected from five major hospitals where AHB was diagnosed. Age and gender composition of the 128 patients was similar to the 294 patients involved in this study (p>0.05 for each). Sixty-eight of the 128 patients were genotyped including 33 (22 men, 11 women) with HBV B2 and 35 (33 men, two women) with HBV C2. In acute resolving hepatitis B, the serum viral load usually declines rapidly due to vigorous immune response.^25^ Seven (two with HBV B2, five with HBV C2) of the 68 patients progressed into chronic infection. HBV genotypes were determined in 107 of the 113 neighbourhood ASCs The proportion of HBV B2 was significantly higher in the patients with AHB than in ASC (fig 3A). The proportion of women in AHB patients with HBV B2 was significantly higher than in those with HBV C2, while the proportion of men in the neighbourhood ASCs with HBV B2 was significantly higher than in those with HBV C2 (fig 3B). Furthermore, the patients with HBV B2 were significantly younger than those with HBV C2 (27.23 (SD 4.28) vs 45.69 (SD 7.02) years, p<0.001) (fig 3C). It is inferred that HBV B2 is transmitted from men to women by sexual activity.

Twenty-five (20 with HBV C2, five with HBV B2) of the 294 patients progressed to chronic infection. The patients with HBV C2 developed chronic infection significantly more often than those infected with HBV B2 (p = 0.013). In multivariate analysis, 25 patients who progressed to chronic infection were compared with 61 HBV-genotyped patients who had not progressed to chronic infection, HBV C2 was found to be a unique independent factor for the chronicification after adjustments with age and sex (table 3).

**Table 3:** Univariate and multivariate regression analyses of the risk factors for chronicification of acute hepatitis B in the patients with hepatitis B virus (HBV) genotyped

	Progression to infection*	Without progression†	OR (95% CI)	AOR (95% CI)
Sex				
Male	19	50		
Female	6	11	1.44 (0.47 to 4.43)	2.61 (0.71 to 9.58)
Age (years)				
⩽40	11	36		
>40	14	25	1.83 (0.72 to 4.69)	0.66 (0.19 to 2.23)
Genotype				
B2	5	31		
C2	20	30	4.13 (1.37 to 12.43)	6.97 (1.59 to 30.63)

*Progression to chronic HBV infection (n = 25). †Without chronic HBV progression (n = 61)

AOR, adjusted odds ratio; CI, confidence interval; OR, odds ratio.

### Serum viral load and HBeAg status

**Figure 4:**
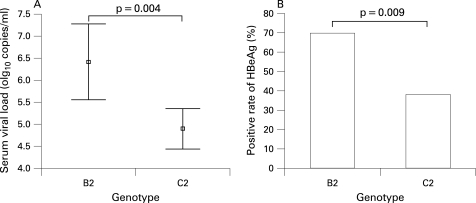
Comparison of serum viral load (A) and hepatitis B e antigen (HBeAg) seropositivity (B) between 20 asymptomatic HBsAg carriers (ASCs) with HBV B2 and 87 ASCs with HBV C2. Serum viral load was transformed into a logarithmic function and expressed as mean with the SD. HBsAg, hepatitis B surface antigen; HBV, hepatitis B virus.

Because sera from the patients with AHB did not suffice for all assays, serum viral load from only one-third of the samples was tested by quantitative PCR. The viral loads varied dramatically. Constant serum viral load was determined in 107 of the 113 ASCs. Serum viral load (6.40 (SD 1.82) log_10_ copies/ml) in ASCs with HBV B2 was significantly higher than the viral load (4.89 (SD 2.13) log_10_ copies/ml) in ASCs with HBV C2 (fig 4A).

In the 68 AHB patients with HBV genotyped, no difference in seropositivity of HBeAg was determined between the patients with HBV B2 (39.39%) and those with HBV C2 (34.29%). In the 25 patients who progressed to chronic infection, seropositivity of HBeAg was 45% and 80% in those with HBV C2 and HBV B2, respectively. No statistical differences in seropositivities of HBeAg were determined between AHB patients and those who progressed to chronic infection, neither in those with HBV B2 nor in those with HBV C2. However, seropositivity of HBeAg in the neighbourhood ASC with HBV B2 (70.00%) was significantly higher than that in those with HBV C2 (37.93%) (fig 4B).

### Phylogenetic relationship of HBV intrafamilial transmission

**Figure 5:**
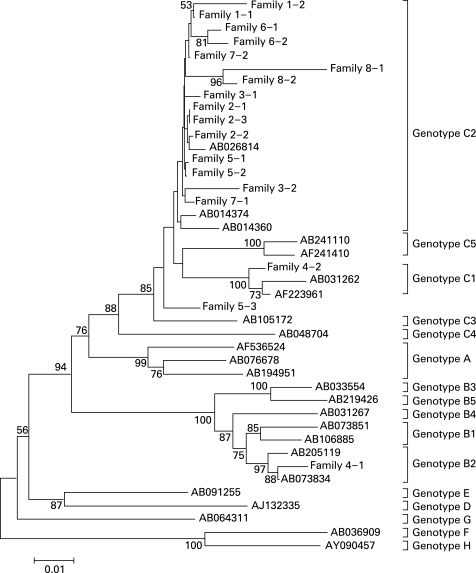
Phylogenetic analysis of the preS and S region of hepatitis B virus (HBV) in eight families (families 1–8) with an index acute hepatitis B (family 1) and the family members. The phylogenetic tree was constructed by using the neighbour-joining component of the MEGA 4.0 computer program. Bootstrap values are shown along each main branch. The sequences of HBV preS and S region obtained from the GenBank database were compared with the same region of HBV isolated in this study. The length of the horizontal bars indicates the number of nucleotide substitutions per site.

In preliminary phylogenetic analysis, household contact with HBV infectants might be supported by corresponding phylogenetic clusters (data not shown). Phylogenetic analysis was then used to determine intrafamilial transmission. Of the 68 AHB patients, eight cases (index: family 1) and their available family members seropositive for HBsAg were included in this assay. Distinct clusters of HBV C2, but not HBV B2, were defined at the family level, including families 2, 5 and 6 and maybe 1 and 8 (fig 5). HBV B2 was identified in the index case of family 4, while this HBsAg positive family member was HBV C1. Thus, household transmission was frequently identified in patients with HBV C2.

## DISCUSSION

In Shanghai, a developed area of China, residents routinely receive HBV screening. Most adults have been tested for HBV markers and have these results documented in their medical records. In this study, apart from serological and clinical chemistry parameters, medical records were used to discriminate newly acquired AHB from acute exacerbation of chronic infection. The patients who did not have clear medical records were excluded. Controls were randomly recruited from the source population from which the cases arose, and matched to the cases by sex and date of birth. Controls were obtained without regard to exposure. To promote participation, standard HBV vaccination was given to those seronegative for HBsAg. A pilot study had been carried out to estimate the sample sizes. This case–control study is reliable.

Undergoing invasive medical procedure, receiving body care and beauty treatments in public places, household contact with HBV-associated diseases and lack of HBV vaccination were current risk factors for adult AHB. The enforced use of disposable syringes for injection has been in place for many years in China. During the survey, we observed that nurses usually did not change their gloves when giving injections or changing bandages after surgery between patients, especially in primary hospitals. These unsatisfactory procedures may contribute to nosocomial transmission of HBV.

Among these risk factors, receiving pedicures in bath centres was emerging. Recently, bath centres which provide pedicure and even hidden sex services have been developing. The health condition of the service professionals and the disinfection situation of the instruments used for the invasive procedures are usually not controlled by local public health agencies. The potential for transmission of infectious diseases exists when receiving manicures and pedicures in urban settings.^26^ Surprisingly, the frequency of eating out was found to be an independent risk factor for AHB. An association of HBV transmission with the consumption of raw seafood has been reported in Korea.^27^ In this present study, we demonstrated that eating out may be an indicator of other exposure-prone behaviours rather than an actual risk factor for AHB (table 2). In China, promiscuous sexual contact is considered immoral and drug injection is illegal. Some participants refused to provide information concerning promiscuous sexual contact or being users of injectable drugs or cohabiting with drug users, resulting in a probable underestimation of these exposures.

In this study, 8.50% of the patients with AHB progressed to chronic infection. Other studies reported rates of 0.2% in Greece, 1% in Japan, 2.7% in Taiwan, 10.4% in Alaskan Inuit, and 8.1% or 12.1% in Germany.^18^ ^28^^–^32 We found different rates of HBV B2 and HBV C2 in AHB and ASC (fig 3A). In ASCs, 81.3% was HBV C2 and 18.7% was HBV B2. The rates were very similar to the figures reported already for chronic HBV patients in this area.^33^ The key result of this study is that HBV C2 was more prone to chronic progression than HBV B2. Gender and age may be determinants of the chronicification because the patients with HBV C2 were much more likely to be older men (fig 3C). In multivariate analysis, HBV C2 was confirmed to be an independent factor for AHB chronicity, while gender and age was not (table 3). Although we did not find any difference in viral load and HBeAg status between the patients with HBV B2 and those with HBV C2, these differences between the neighbourhood ASC with HBV B2 and those with HBV C2 were apparent (fig 4). High concentrations of HBV DNA were found in saliva, semen, nasopharyngeal fluid, urine and tears of ASCs with high viral load,^34^^–^36 indicating that exposure to these fluids may lead to infection. The higher virus load may have contributed to the more frequent transmission of HBV B2. However, it did not cause a higher rate of chronicity. Higher rate of persistence was not due to higher viral DNA concentrations of HBV C2 in ASC, in contrast to HBV B2 (fig 4A). HBeAg is involved in evading the immune response.^37^ ^38^ Thus, HBeAg expression was expected to be associated with transition to chronicity. Surprisingly, there were no differences in HBeAg expression in our AHB patients with or without resolution for both HBV B2 and HBV C2. Thus, the only factor identified as a risk factor for chronicification of AHB in our study remains HBV C2.

Interestingly, AHB patients with HBV B2 were much younger, with a higher proportion of women than those with HBV C2. ASC with HBV B2 showed a higher viral load, higher HBeAg status, and higher proportion in men than those with HBV C2. This molecular evidence indicates that heterosexual activity was the most likely transmission route of HBV B2 although this risk factor was not revealed by the field epidemiological study.

Phylogenetic analysis showed that distinct clusters of HBV C2 were defined at the family level. Household contact with HBV-positive family members is one of the most likely transmission routes of HBV C2. Interestingly, we found a family member with HBV C1, which is frequently found in southern China. In Shanghai and its surrounding area, HBV C1 is very rare.^15^

Major risk factors suggested in this study should be useful in conducting public health interventions for the prevention and control of AHB. If we take into account all the data from this study, an important academic conclusion is that there are inherent – as yet unknown – properties encoded in HBV C2 that lead to higher persistence after acute infection. The identification of the properties of HBV C2 that lead to immune evasion may improve our understanding of the viral life cycle and enable strategies to break immune tolerance induced by HBV.
